# YY1-modulated long non-coding RNA SNHG12 promotes gastric cancer metastasis by activating the miR-218-5p/YWHAZ axis

**DOI:** 10.7150/ijbs.58921

**Published:** 2021-04-12

**Authors:** Tianqi Zhang, Maneesh Kumarsing Beeharry, Zhenqiang Wang, Zhenggang Zhu, Jianfang Li, Chen Li

**Affiliations:** Department of General Surgery, Shanghai Key Laboratory of Gastric Neoplasms, Shanghai Institute of Digestive Surgery, Ruijin Hospital, Shanghai Jiao Tong University School of Medicine, Shanghai 200025, China

**Keywords:** gastric cancer, SNHG12, miR-218-5p, YWHAZ, metastasis

## Abstract

Long non-coding RNA (lncRNA) small nucleolar RNA host gene 12 (SNHG12) plays important roles in the pathogenesis and progression of cancers. However, the role of SNHG12 in the metastasis of gastric cancer (GC) has not yet been thoroughly investigated. In the present study, we demonstrated that SNHG12 was upregulated in GC tissues and cell lines. In addition, the expression level of SNHG12 in GC samples was significantly related to tumor invasion depth, TNM stage and lymph node metastasis and was associated with disease-free survival (DFS) and overall survival (OS) in GC patients. *In vivo* and *in vitro* assays indicated that SNHG12 promotes GC metastasis and epithelial-mesenchymal transition (EMT). Bioinformatics and mechanistic analyses revealed that SNHG12 can directly target miR-218-5p to regulate YWHAZ mRNA, forming an SNHG12/miR-218-5p/YWHAZ axis and decreasing the ubiquitination of β-catenin. In addition, SNHG12 stabilizes CTNNB1 mRNA by binding with HuR, thus activating the β-catenin signaling pathway. Further analysis also revealed that the transcription factor YY1 negatively modulates SNHG12 transcription. In conclusion, SNHG12 is a potential prognostic marker and therapeutic target for GC. Negatively modulated by YY1, SNHG12 promotes GC metastasis and EMT by regulating the miR-218-5p/YWHAZ axis and stabilizing CTNNB1 via activation of the β-catenin signaling pathway.

## Introduction

Gastric cancer (GC) is the 5^th^ most common malignancy and 3^rd^ leading cause of cancer deaths in the world 1. The prognosis of GC remains poor due to tumor metastasis and recurrence and other potential underlying factors, including the heterogeneous functions of key genes and crosstalk between signaling molecules and regulatory networks involved in the initiation, progression and invasion of GC 2. GC metastasis is a complex and multistep process, and epithelial-mesenchymal transition (EMT), which is characterized by decreased expression of cell adhesion molecules (such as E-cadherin) and increased expression of mesenchymal markers (such as vimentin), plays an important role in tumor metastasis 3. Reduced E-cadherin expression results in increased stability and cytoplasmic accumulation of β-catenin, which can localize in the nucleus to form a transcriptional complex with T-Cell Factor/Lymphoid Enhancer Factor (TCF/LEF), thus promoting the transcription of some genes, such as snail 3, 4.

Recent findings have shown that long non-coding RNAs (lncRNAs) play important roles in a wide range of physiological and pathophysiological processes and act as tumor promotors or tumor suppressors in tumorigenesis and cancer metastasis 5-7. The lncRNA small nucleolar RNA host gene 12 (SNHG12) is located on chromosome 1 in the 1p35.3 region and was first reported to be significantly upregulated in endometrial cancer in humans 8. Recent studies have shown that the altered expression of SNHG12 can promote metastasis in different tumors via diverse pathways 9; for instance, SNHG12 has been shown to promote metastasis in GC by sponging miR-320 and miR-16 or activating the PI3K/AKT pathway 10-12. Nevertheless, the roles of SNHG12 in other key EMT and metastasis pathways still need deeper exploration.

miR-218-5p is a well-known tumor suppressor that is also recognized as a predictor of metastasis in various types of cancers 13, 14. It was demonstrated that miR-218-5p could suppress the GC cell cycle through the CDK6/cyclin D1/E2F1 axis in a feedback loop 15. Tyrosine 3 monooxygenase/tryptophan 5-monooxygenase activation protein zeta, also named YWHAZ or 14-3-3ζ, is a key modulator of the β-catenin signaling pathway and is closely associated with tumorigenesis and cancer metastasis 16. In the preliminary evaluation of this study, we obtained bioinformatics evidence of possible interactions among SNHG12, miR-218-5p and YWHAZ, and we therefore investigated the associated molecular mechanisms in GC metastasis.

Yin Yang 1 (YY1) belongs to the GLI-Kruppel class of zinc-finger proteins and acts as a transcription repressor or activator to regulate a series of biological processes, such as embryogenesis, cellular proliferation, differentiation, and tumorigenesis. Nevertheless, its role in the regulation of tumor progression remains controversial 17.

Therefore, in the present study, we profiled SNHG12, miR-218-5p, YWHAZ, CTNNB1 and YY1 expression in GC tissues and cells and investigated the role and underlying mechanisms of SNHG12 in GC metastasis and EMT.

## Materials and methods

### GC patients and tissue specimens

A total of 54 GC tissues and the corresponding adjacent noncancerous epithelial tissues were obtained from GC patients undergoing radical surgery at Ruijin Hospital affiliated to Shanghai Jiao Tong University School of Medicine from 2015 to 2019. The patients did not undergo radiotherapy or chemotherapy prior to surgery. All patients were independently diagnosed histologically by two experienced pathologists and staged according to the TNM staging system of the American Joint Committee on Cancer (AJCC 7^th^ ed., 2010). All tissue samples were immediately frozen in liquid nitrogen after resection from GC patients and stored at -80 °C for further analysis. The acquisition of the tissues was approved by the Ruijin Hospital Ethics Committee (Ethics approval number: 2017-6).

### Cell lines and culture conditions

Four human GC cell lines (AGS, MGC-803, SGC-7901 and HS-746T), HEK-293T and the non-malignant gastric mucosal epithelial cell line GES-1 were purchased from the Cell Bank of the Chinese Academy of Sciences (Shanghai, China). Cells were cultured in DMEM (Gibco, BRL, San Francisco, USA) medium supplemented with 10% fetal bovine serum (HyClone, Carlsbad, CA, USA) and 5 μg/ml penicillin and streptomycin maintained in a humidified atmosphere at 37 °C in 5% CO_2_.

### RNA extraction and quantitative reverse transcription PCR (qRT-PCR)

Total RNA was isolated from patient tissues and cultured cells using TRIzol reagent (Vazyme, Nanjing, China) according to the manufacturer's instructions. Cytoplasmic and nuclear RNA purification were conducted according to the protocol in the kit purchased from Norgen (#21000, 37400). RNA was reverse transcribed into cDNA using HiScript III RT SuperMix for qPCR (Vazyme, Nanjing, China), while microRNA (miRNA) was reverse transcribed into cDNA using the miRNA First Strand cDNA Synthesis kit (Sangon Biotech, Shanghai, China). cDNA was quantified by RT-PCR and SYBR Green (Vazyme, Nanjing, China) was detected using an Applied Biosystems 7500 instrument. GAPDH, U6 and ACTB were used as internal controls. The experiments were performed in triplicate and repeated three times. The primers used in the study are listed as follows:

### Lentivirus production, small interfering RNA (siRNA), plasmid and cell transfection

Lentivirus-containing short hairpin RNA (shRNA) targeting SNHG12 was purchased from OBiO (Shanghai, China), the sequences are as follows: sh-SNHG12-1: CcggGCTGTCCTCATTTGTGACTTTCAAGAGAAGTCACAAATGAGGACAGCTTTTTTg, sh-SNHG12-2: CcggCCTATGGAGTTGGGACAATTTCAAGAGAATTGTCCCAACTCCATAGGTTTTTTg. And the pCDH-CMV-Human vector for SNHG12 overexpression was purchased from Allwin (Shanghai, China). miR-218-5p mimics, miR-218-5p inhibitors, and negative control (NC) oligonucleotides were obtained from GenePharma (Shanghai, China). SiRNAs for YY1, YWHAZ and HuR were obtained from Sangon Biotech (Shanghai, China), sequences are listed as follows: si-YY1 sense (5'-3'): CCAAACAACUGGCAGAAUUTT, antisense (5'-3'): AUUCUGCCAGUUGUUUGGTT; si-HuR sense (5'-3'): GCGUUUAUCCGGUUUGACAtt, antisense (5'-3'): UGUCAAACCGGAUAAACGCtt; si-YWHAZ sense (5'-3'): GATGACATGGCAGCCTGCATGAAGT. GC cells were transfected with the abovementioned oligonucleotides and plasmids using Lipofectamine 2000 (Invitrogen) according to the manufacturer's protocol.

### Cell migration

Cell migration ability was measured using Transwell chambers (8-μm pore size; Corning Costar, Cambridge, MA, USA). For the Transwell assay, 5×10^4^ cells suspended in serum-free DMEM were seeded into the upper chamber. The lower chamber contained DMEM supplemented with 5% serum. After 10h of incubation, the filters were fixed in methanol and stained with 0.1% crystal violet. The upper faces of the filters were gently abraded, and the lower faces containing cells that had migrated across the filters were imaged, and the cells were counted under a microscope. These experiments were performed in triplicate and repeated three times.

### Western blot

Total proteins from cells were extracted using RIPA buffer supplemented with protease inhibitors and phosphatase inhibitors. Western blot was conducted as the routine protocol. Supernatants derived from cell extracts were separated on a 10% SDS-PAGE gel and then transferred to PVDF membranes. PVDF membranes were blocked in 5% bovine serum albumin (BSA) for 1 h and then incubated with diluted primary antibodies at 4 °C overnight. The primary antibodies used in this assay included antibodies against E-cadherin (ABclonal, #A3044), vimentin (Cell Signaling Technology, #5741S), N-cadherin (Proteintech, #22018-1-AP), β-catenin (Cell Signaling Technology, #8480S), Snail (Cell Signaling Technology, #3879S), YWHAZ (ABclonal, #A13370), GAPDH (Proteintech, #60004-1-Ig), and lamin B (Proteintech, #12987-1-AP). These experiments were performed in triplicate and repeated three times.

### Fluorescence *in situ* hybridization (FISH) and *in situ* hybridization (ISH)

The FISH assays of GC cells and ISH assays of tissues were conducted according to a method described previously 18, 19. The RNA probes targeting SNHG12 were designed and synthesized by Servicebio, the sequence is listed as followed: SNHG12-H 5'-GCTCCTCCGTGCCACATTCACCACCATCTC -3'.

### Immunohistochemistry (IHC)

IHC assays of tissues were performed as previously described 18. Briefly, tumor tissues from mice were embedded and sectioned and then incubated with antibodies against N-cadherin (Proteintech, #22018-1-AP) and E-cadherin (ABclonal, #A3044). After washing the samples with PBS, the samples were incubated with secondary antibody, followed by DAB treatment. The staining intensity was graded into four ranges (intensity score): no staining (0), light brown staining (1), brown staining (2) and dark brown staining (3). The number of positively staining GC cells was divided into four ranges (percentage score): < 5% (0), 5-25% (1), 26-50% (2), 51-75% (3), > 75% (4). The final staining score was calculated using the formula: overall score = intensity score × percentage score. A final score 0-7 was defined as low expression, and >8 as high expression. The scores were evaluated by two independent, board-certified pathologists in an unbiased manner.

### Luciferase reporter and TOPFlash/FOPFlash reporter assays

Luciferase reporter plasmids carrying a wild-type (WT) or mutated (MUT) 3'-UTR of SNHG12 and a WT or MUT 3'-UTR of YWHAZ were purchased from Public Protein/Plasmid Library (Nanjing, China). The above plasmids were transfected into GC cells along with the miR-218-5p mimics using Lipofectamine 2000. After transfection (36-48 h), the cells were lysed, and luciferase activity was measured with the Dual-Luciferase Reporter Assay system (Promega). The TOPFlash/FOPFlash reporter assay was employed according to the instructions of the TCF Reporter Plasmid Kit (Millipore). These experiments were performed in triplicate and repeated three times.

### Co-immunoprecipitation (Co-IP) and immunoprecipitation (IP)

Co-IP and IP were conducted using the IP/Co-IP kit (ABsin, #abs955) according to the manufacturer's instructions. The primary antibodies used in this assay included antibodies against β-catenin (ABclonal, #A11932), YWHAZ (Proteintech, #14881-1-AP), ubiquitin (ABclonal, #A19686), and β-tubulin (ABclonal, #A12289). These experiments were performed in triplicate and repeated three times.

### Chromatin immunoprecipitation (Ch-IP) assay

Ch-IP assays were performed using the EZ-Magna Ch-IP Kit (Millipore 17-10086), as previously described 20. The primary antibody used in this assay was an antibody against YY1 (Proteintech, #22156-1-AP). These experiments were performed in triplicate and repeated three times. The primers used in this assay are listed as follows:

### RNA binding protein immunoprecipitation (RIP)

RIP was performed using the EZ-magna RIP kit (Millipore 17-700), and the antibodies used in this assay included antibodies against Ago2 (Abcam, #ab32381) and HuR (Cell Signaling Technology, #12582S). These experiments were performed in triplicate and repeated three times. The primers used in this assay are listed as follows:

### RNA stability assays

GC cells were treated with actinomycin D at a concentration of 5 μg/ml. The cells were harvested at 0, 3, 6, and 9 h after the actinomycin D treatment, and RNA was extracted with TRIzol reagent. Then, the mRNA levels were detected by qRT-PCR.

### *In vivo* metastasis assays

Four-week-old female immunodeficient BABL/c nude mice were purchased and maintained under specific pathogen-free conditions. Mice were randomly divided into two groups with five mice for per group. All experiments were performed in accordance with the official recommendations of the Chinese Animal Community. The acquisition of the tissues was approved by the Ruijin Hospital Ethics Committee. MGC-803 cells (2×10^6^) stably expressing sh-SNHG12 or sh-NC were separately injected into the abdomen of mice, and the body weight of the mice was measured and recorded every 3 days. After 1 month, the mice were sacrificed, and abdominal tumors were dissected for ISH and IHC assays. The experimenters responsible for the animal procedures were blinded to the grouping of the animals.

### Statistical analysis

All statistical analyses were conducted using SPSS 23.0 (SPSS, Chicago, IL, USA) or GraphPad Prism V8 (GraphPad Prism, Inc., La Jolla, CA, USA). Each experiment was performed at least in triplicate, and data are presented as the mean ± SD of three independent experiments. Student's t-test or one-way ANOVA was used to compare the means of two or three groups. P values less than 0.05 were considered statistically significant.

## Results

### Overexpression of SNHG12 is associated with advanced GC stage and indicates poor prognosis in GC patients

**Figure 1:**
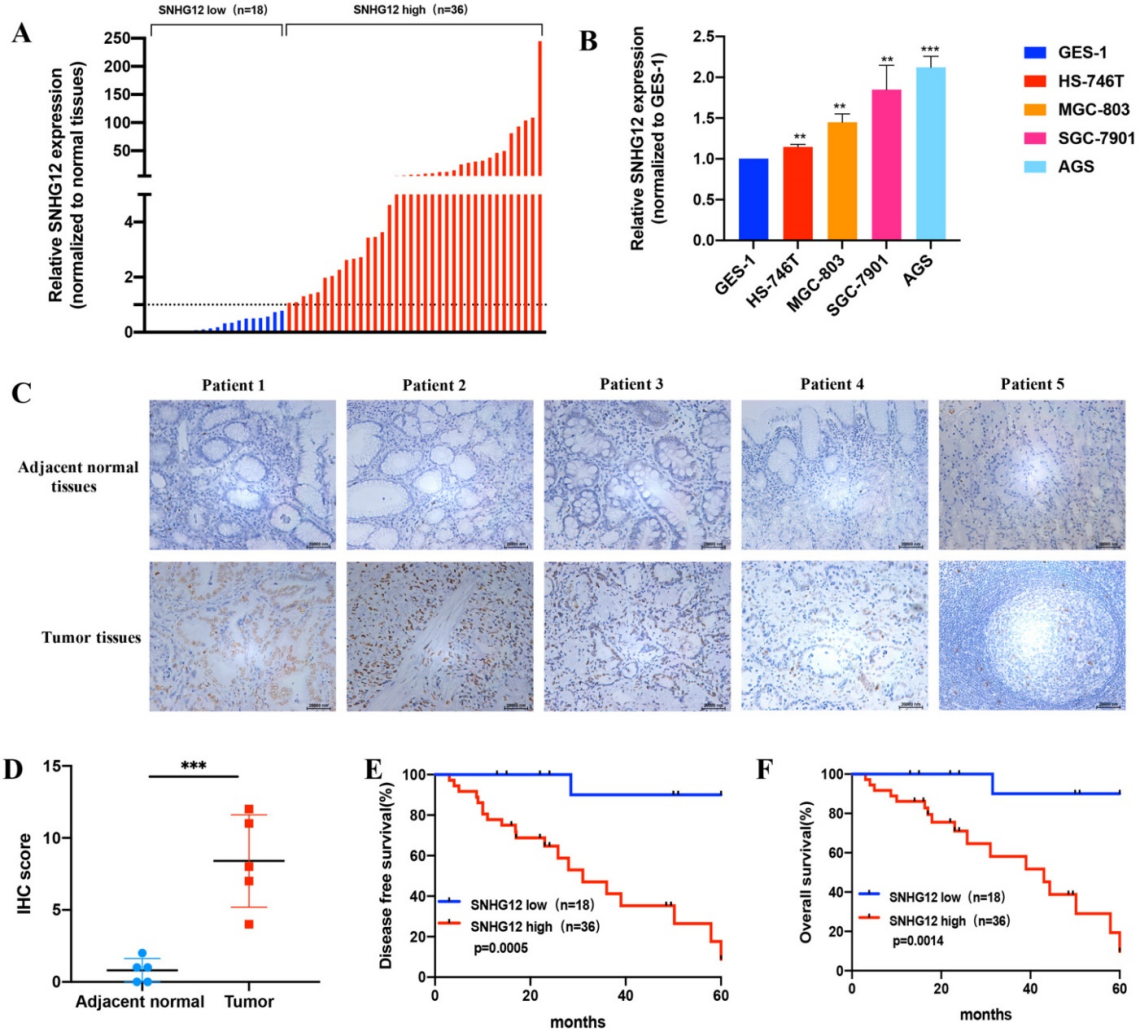
** SNHG12 is overexpressed in GC tissues and cell lines and indicates poor prognosis in GC. (A)** SNHG12 expression in 54 pairs GC tissues and corresponding adjacent non-cancerous epithelial tissues. **(B)** qRT-PCR assays show the relative SNHG12 expression in GC cell lines normalized to GES-1. **(C, D)** ISH assays showing SNHG12 expression in normal tissues and tumor tissues, magnification ×200. Histogram show the ISH scores of SNHG12 in tumor tissues and adjacent non-cancerous epithelial tissues. **(E, F)** Kaplan-Meier analysis showing the 5-year DFS and OS of GC patients with high SNHG12 expression or low SNHG12 expression. Scale bar, 20 µm. Significant results were presented as **P<0.01, ***P<0.001.

The expression pattern of SNHG12 in human GC samples and GC cell lines was determined by qRT-PCR assays. We detected the relative expression of SNHG12 in 54 cases of GC tissues and the corresponding adjacent noncancerous epithelial tissues. As shown in Figure 1A, SNHG12 was markedly upregulated in 66.7% (36/54) of GC tissues compared to adjacent noncancerous epithelial tissues (The ratio of SNHG12 expression in GC tissue sample to the corresponding adjacent noncancerous epithelial tissues was used to define the SNHG12 expression status in the sample: if the ratio was above 1, it defined high SNHG12 expression in GC while vice versa).

When expression was normalized to that in the GES-1 normal gastric epithelial cell line, SNHG12 was found to be significantly highly expressed in HS-746T, MGC-803, SGC-7901, and AGS cells (Figure 1B). Based on their overall SNHG12 expression characteristics, MGC-803 and AGS cells were selected for further *in vitro* mechanistic experiments. ISH assays showed that the intensity of SNHG12 staining in GC tissues was much higher than that in the corresponding adjacent noncancerous epithelial tissues (Figure 1C, D).

To further establish the clinical significance of the difference in the expression levels of SNHG12, we analyzed the correlation between SNHG12 and the clinicopathological characteristics of the 54 GC patients, and we discovered that higher SNHG12 expression was related to depth of tumor invasion, extent of lymph node metastasis and the TNM stage in GC patients (Table 1). Kaplan-Meier survival analysis indicated that patients with high SNHG12 expression had poorer survival outcomes than those with low SNHG12 expression. The 5-year disease-free survival (DFS) rates of the high and low SNHG12 expression groups were 44.4% vs. 94.4%, respectively (p=0.0005) (Figure 1E), and the 5-year overall survival (OS) rates were 52.8% and 94.4%, respectively (p=0.0014) (Figure 1F). Moreover, univariate analysis indicated that TNM stage and SNHG12 expression were predictors of DFS and OS in GC patients (all p<0.05, Table 2, 3). Multivariate analysis showed that SNHG12 was an independent prognostic factor of DFS and OS (all p<0.05, Table 2, 3).

### SNHG12 promotes GC cell migration and EMT *in vitro* and *in vivo*

**Figure 2:**
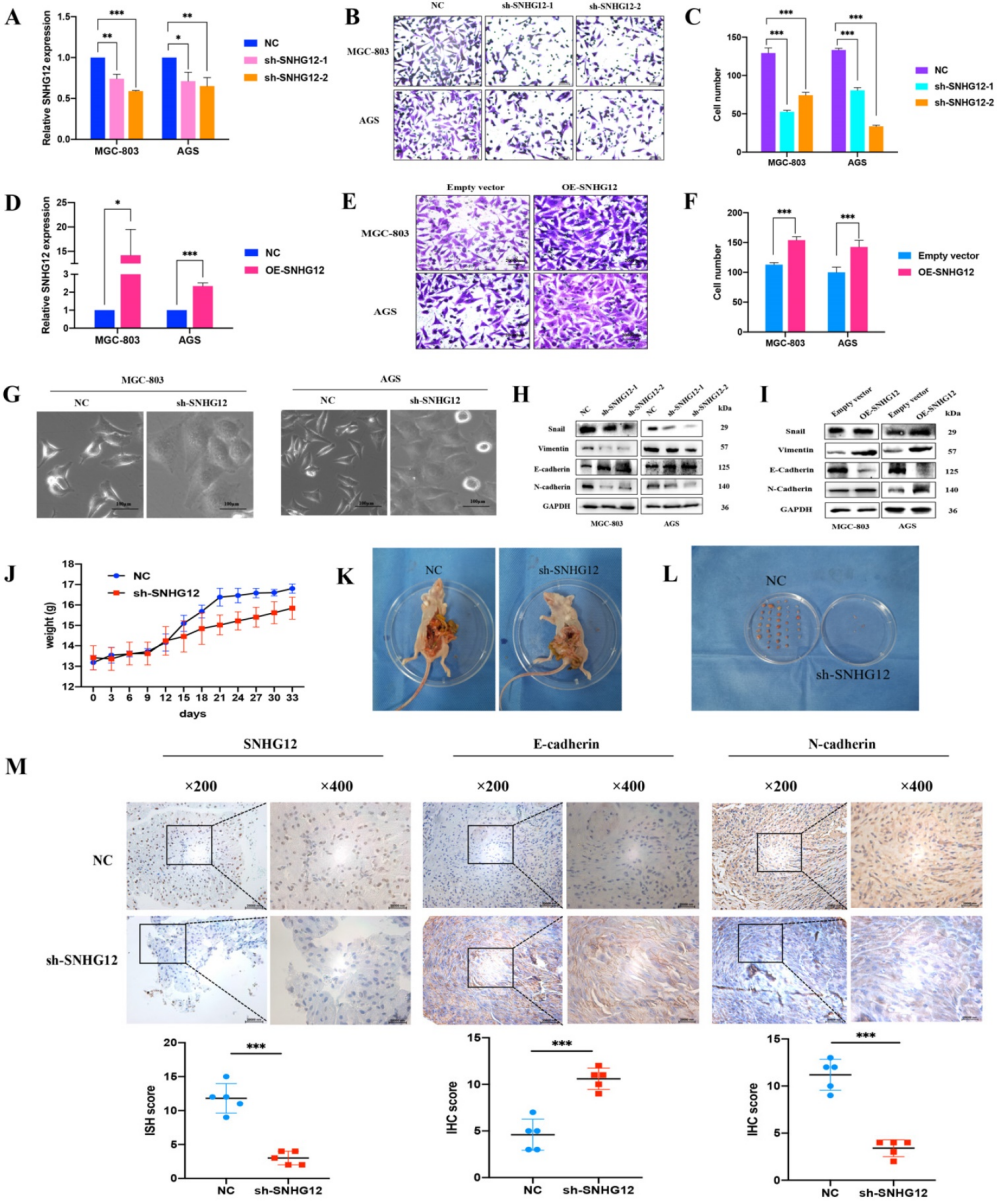
** SNHG12 promotes GC migration and EMT *in vitro* and vivo. (A)** The efficiencies of SNHG12 knockdown in MGC-803 and AGS cells detected by qRT-PCR. **(B, C)** Transwell assays showing the effects of SNHG12 knockdown on GC cell migration. **(D)** The efficiencies of SNHG12 overexpression in MGC-803 and AGS cells detected by qRT-PCR. **(E, F)** Transwell assays showing the effects of SNHG12 overexpression on GC cell migration. Magnification ×200, Scale bar 20 µm. Significant results were presented as *P<0.05, **P<0.01, ***P<0.001. **(G)** Morphological change of the cells with stable SNHG12 knockdown (sh-SNHG12) compared with mock control cells (NC). Magnification ×200, Scale bar 100 µm.** (H, I)** WB assays exhibit change of EMT markers among stable SNHG12 knockdown cells (sh-SNHG12-1 and (sh-SNHG12-2), stable SNHG12 overexpressed cells (OE-SNHG12) and mock control cells (NC). **(J)** Body weight of the animal subjects were recorded every 3 days for 1 month. **(K, L)** Obvious metastatic formations in the control group (NC) as compared with the SNHG12 knockdown group (sh-SNHG12). **(M)** ISH and IHC assays showing SNHG12, N-cadherin, and E-cadherin intensity between NC group samples and sh-SNHG12 group samples. Magnification ×200 and ×400, Scale bar 20 µm. Histogram show the ISH scores of SNHG12 and IHC scores of N-cadherin and E-cadherin in NC group samples and sh-SNHG12 group samples.

Loss-and-gain assays were conducted in the GC cell lines MGC-803 and AGS using shRNA and the pCDH-CMV-Human vector, and the SNHG12 knockdown and overexpression efficiencies were evaluated by qRT-PCR (Figures 2A, D). Transwell assays indicated that knockdown of SNHG12 significantly suppressed GC cell migration (Figures 2B, C), while overexpression of SNHG12 promoted GC cell migration (Figures 2E, F).

While investigating the influence of SNHG12 regulation on EMT in GC cells, we found that knockdown of SNHG12 induced morphological changes in GC cells: the cells changed from a spindle-shaped mesenchymal appearance to a cobble stone-like, spherical appearance (Figure 2G). The western blot results showed that knockdown of SNHG12 decreased the expression of the mesenchymal markers N-cadherin and vimentin and the EMT-related transcription factor Snail, and increased the expression of the epithelial marker E-cadherin; on the other hand, overexpression of SNHG12 induces the opposite effects (Figures 2H, I).

To further validate the metastatic potential of SNHG12 *in vivo*, a nude mouse metastasis model was constructed. After establishing the model, significant differences were noted in the body weight of the mice: the normal control (NC) mice showed significant weight gain compared with the sh-SNHG12 mice, indicating possible mass formation (Figure 2J). After 1 month of observation, the mice were sacrificed, and the anatomical dissection findings were as follows: in the NC mice, there was notable inflammatory adhesion in the mesentery with nodular formations in the mesentery and intestinal surface (approximately 6 to 8 masses in each mouse), while in the sh-SNHG12 mice, the abdominal cavity was clear with occasional nodular findings (3 masses found in 3 of the mice) (Figures 2K, L). ISH and IHC analyses of the masses showed that the SNHG12 and N-cadherin intensities were higher in the NC group than in the sh-SNHG12 group, while the E-cadherin intensity was higher in the sh-SNHG12 group (Figure 2M).

### SNHG12 acts as a competitive endogenous RNA for miR-218-5p to regulate YWHAZ expression in GC cells

**Figure 3:**
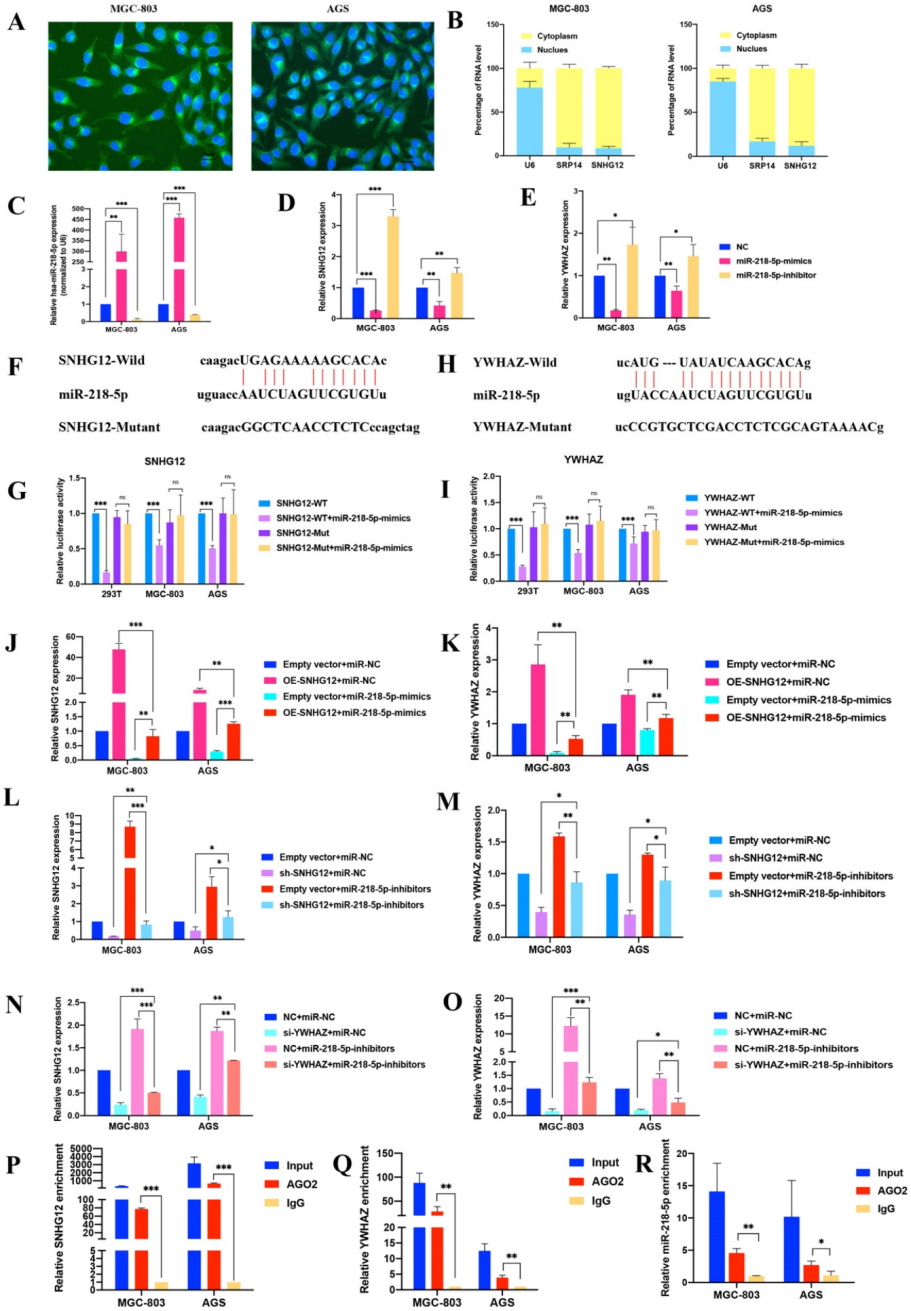
** SNHG12 acts as a ceRNA for miR-218-5p to regulate YWHAZ expression in GC cells. (A, B)** FISH assays and cytoplasmic and nuclear RNA purification assays indicate that SNHG12 is located in GC cell cytoplasm. **(C, D, E)** qRT-PCR assays showing relative expression of miR-218-5p, SNHG12 and YWHAZ in GC cells transfected with miR-218-5p mimics or inhibitors. **(F, G)** Luciferase assays revealed the interaction between miR-218-5p and SNHG12. **(H, I)** Luciferase assays revealed the interaction between miR-218-5p and YWHAZ. **(J, K, L, M, N, O)** qRT-PCR assays indicated the relative expression of SNHG12 and YWHAZ in GC cells transfected with miR-218-5p mimics or pCDH-CMV-SNHG12 and sh-SNHG12 or si-YWHAZ or miR-218-5p inhibitors. **(P, Q, R)** RIP assays indicated the binding of SNHG12, miR-218-5p, or YWHAZ with Ago2. Significant results were presented as *P<0.05, **P<0.01, ***P<0.001. Magnification ×200, Scale bar 50 µm. Magnification ×400, Scale bar 20 µm.

The FISH assays showed that SNHG12 was mainly located in the cytoplasm (Figure 3A). Cytoplasmic and nuclear RNA purification assays further confirmed that the majority of SNHG12 transcripts were located in the cytoplasm instead of the nucleus (Figure 3B). This result suggests that SNHG12 mainly exerts its function at the post-transcriptional level and may sponge miRNAs to regulate downstream molecules.

Bioinformatics analysis via the Starbase and miRcode databases indicated that miR-218-5p has binding sites complementary to the 3'-UTR of SNHG12, suggesting the direct sponging of miR-218-5p by SNHG12 (Additional File 1: Figure S1). Further bioinformatics analysis via the TargetScan Human 7.2 and Starbase databases revealed that YWHAZ could be a downstream gene of SNHG12 and miR-218-5p (Additional File 1: Figure S2).

Modulation of miR-218-5p expression in the MGC-803 and AGS cell lines was achieved by treatment with miR-218-5p mimics for overexpression and miR-218-5p inhibitors for suppression. Compared with the miR-NC groups, the expression of SNHG12 and YWHAZ was suppressed in the miR-218-5p mimic group but increased in the inhibitor group (Figures 3C-E). We further investigated whether miR-218-5p could directly bind to the 3'-UTRs of SNHG12 and YWHAZ, and dual-luciferase reporter assays indicated a significant reduction in luciferase activities after co-transfection of miR-218-5p-mimics and a WT SNHG12 reporter vector or a WT YWHAZ reporter vector, but this reduction was not observed upon transfection with a 3'-UTR-mutant SNHG12 reporter vector or 3'-UTR-mutant YWHAZ reporter vector (Figures 3F-I). To further elucidate the relationship between SNHG12, miR-218-5p and YWHAZ, pCDH-CMV-SNHG12 or miR-218-5p mimics were transfected into the MGC-803 and AGS cells. qRT-PCR assays indicated that the expression of SNHG12 and YWHAZ was significantly increased and decreased by transfection with pCDH-CMV-SNHG12 and miR-218-5p mimics, respectively. On the other hand, when MGC-803 and AGS cells were co-transfected with pCDH-CMV-SNHG12 and miR-218-5p mimics, both of the above effects were reversed (Figures 3J, K). Likewise, when sh-SNHG12-2 or si-YWHAZ, or miR-218-5p inhibitors were transfected into MGC-803 and AGS cells, the relative expression of SNHG12 and YWHAZ was decreased or increased, respectively. When sh-SNHG12-2 or si-YWHAZ and miR-218-5p inhibitors were co-transfected into the MGC-803 and AGS cells, both of the above effects were reversed (Figures 3L-O). RIP assays specific for Ago2, a component of the RNA-induced silencing complex (RISC), were conducted, and the results revealed that SNHG12, miR-218-5p and YWHAZ could bind to Ago2 (Figures 3P-R). These results suggested that SNHG12 competes with YWHAZ to interact with miR-218-5p-containing RISCs.

### SNHG12 activates β-catenin by reducing its ubiquitination-based degradation and stabilizing CTNNB1 mRNA

**Figure 4:**
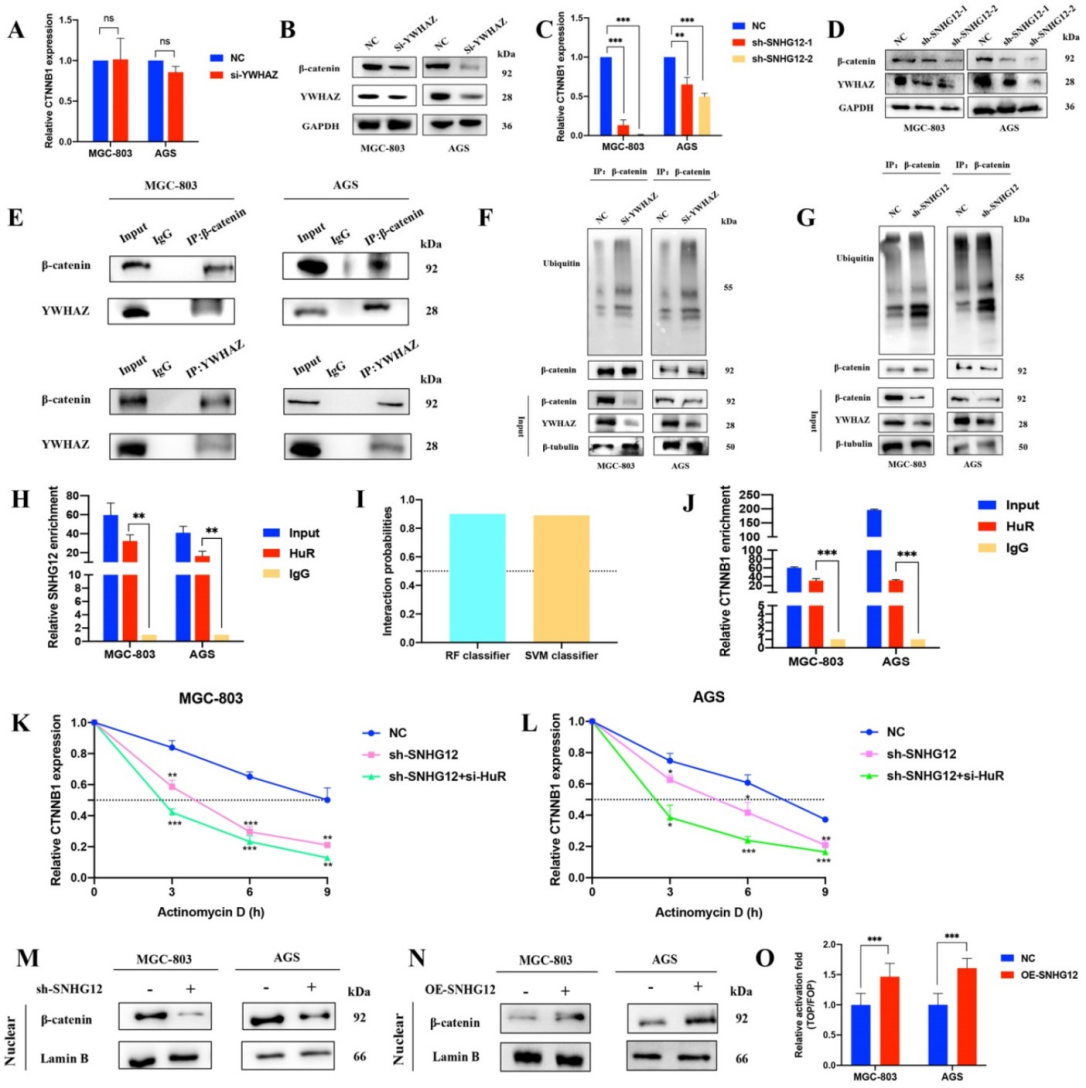
**SNHG12 increases the expression of β-catenin via YWHAZ stabilizing β-catenin and stabilizing CTNNB1 mRNA. (A, B)** The RNA and protein expressions of β-catenin after YWHAZ knockdown were verified by qRT-PCR and WB. **(C, D)** The RNA and protein expressions of β-catenin after SNHG12 knockdown were verified by qRT-PCR and WB.** (E)** Co-IP assays showing the interaction between YWHAZ and β-catenin. **(F, G)** Ubiquitination changes after knockdown YWHAZ and SNHG12 tested by IP in GC cells. **(H)** RIP assays showing SNHG12 binding with HuR. **(I)** Interaction probabilities between HuR and CTNNB1 predicted by RPIseq online tool. **(J)** RIP assays showing CTNNB1 binding with HuR. **(K, L)** The stability of CTNNB1 after knockdown SNHG12 or HuR.** (M, N)** The nuclear expression of β-catenin after SNHG12 knockdown and overexpression was tested by western blotting. **(O)** Luciferase assays showing the effects on TOP/FOP reporter activity in MGC-803 and AGS cells with SNHG12 overexpression. Significant results were presented as *P<0.05, **P<0.01, ***P<0.001. No significantly differences were presented as ns.

The Wnt/β-catenin signaling pathway has a well-established role in cancer cell invasion and EMT. The protein encoded by YWHAZ, 14-3-3ζ, can interact with β-catenin to increase its expression by decreasing its ubiquitination. According to previous studies, we focused on the mechanism by which SNHG12 regulates β-catenin signaling activity via 14-3-3ζ. As shown in Figures 4A, B, YWHAZ knockdown led to a decrease in β-catenin at the protein level but no obvious change at the RNA level. In line with this, SNHG12 knockdown resulted in a decrease in β-catenin at both the RNA and protein levels (Figures 4C, D). Co-IP assays validated the interaction of the YWHAZ protein and β-catenin in GC cells (Figure 4E), and IP assays proved that the ubiquitination level was increased in the YWHAZ or SNHG12 knockdown group, compared with the mock control group (Figures 4F, G).

To further investigate the mechanisms by which SNHG12 regulates CTNNB1 mRNA, we first hypothesized that SNHG12 could stabilize CTNNB1 mRNA by binding to some RNA-binding proteins (RBPs). HuR is a popular tumor-related RBP, and RIP assays were conducted to verify that SNHG12 could bind with HuR (Figure 4H). Using the RNA-Protein Interaction Prediction (RPISeq) (http://pridb.gdcb.iastate.edu/RPISeq/) online tool, the interaction probabilities of CTNNB1 mRNA (encoding the β-catenin protein) and HuR were high: the RF classifier and SVM classifier scores were 0.9 and 0.89, respectively (Figure 4I). RIP assays further validated that CTNNB1 could bind to HuR in MGC-803 and AGS cells (Figure 4J). RNA stability assays were conducted, and demonstrated that the half-life of CTNNB1 mRNA was significantly reduced in the SNHG12 or HuR knockdown group compared with the NC group (Figures 4K, L).

Moreover, nuclear expression of β-catenin was detected in cells with SNHG12 knockdown and overexpression. Western blot assays indicated that the nuclear expression of β-catenin dramatically decreased when SNHG12 was knocked down, while β-catenin nuclear expression increased when SNHG12 was overexpressed (Figures 4M, N). TOPFlash and FOPFlash reporters were constructed to verify whether SNHG12 expression modulated the activation of the β-catenin pathway, and as expected, the overexpression of SNHG12 in MGC-803 and AGS cells resulted in a remarkable increase in TOP/FOP reporter activity (Figure 4O), suggesting activation of β-catenin-dependent transcription.

### The SNHG12/miR-218-5p/YWHAZ axis positively regulates GC cell metastatic potential via the β-catenin pathway

**Figure 5:**
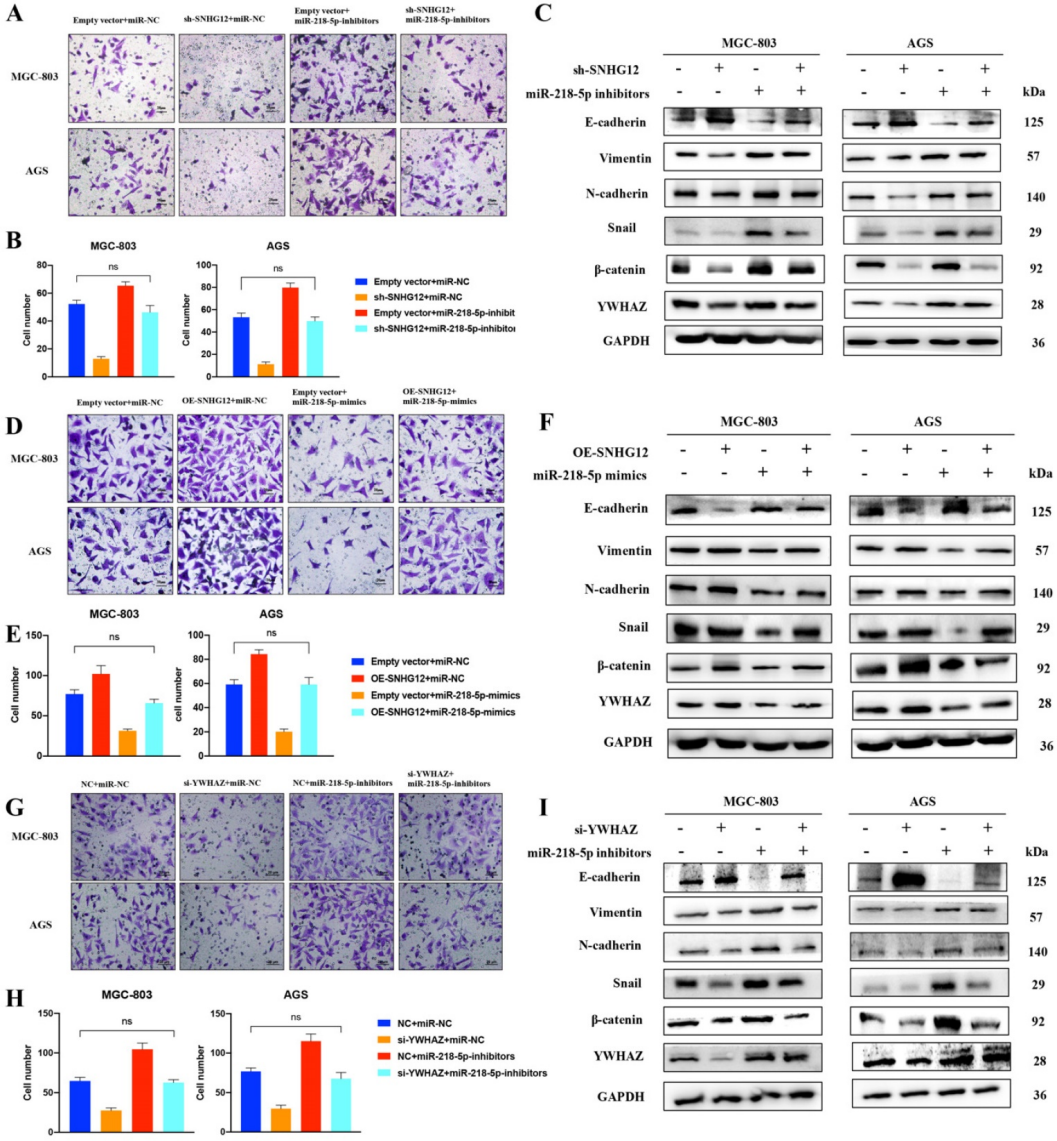
** SNHG12/miR-218-5p/YWHAZ axis positively regulates GC cell metastatic potential via β-catenin pathway. (A, B)** Transwell assays transfected with pCDH-SNHG12 or miR-218-5p mimics or both in MGC-803 and AGS. **(C)** WB assays showing the expression of EMT-related proteins, YWHAZ encoded protein and β-catenin transfected with pCDH-SNHG12 or miR-218-5p mimics or both in MGC-803 and AGS. **(D, E)** Transwell assays transfected with sh-SNHG12 or miR-218-5p inhibitors or both in MGC-803 and AGS. **(F)** WB assays showing the expression of EMT-related proteins, YWHAZ encoded protein and β-catenin transfected with sh-SNHG12 or miR-218-5p inhibitors or both in MGC-803 and AGS. **(G, H)** Transwell assays transfected with si-YWHAZ or miR-218-5p inhibitors or both in MGC-803 and AGS. **(I)** WB assays showing the expression of EMT-related proteins, YWHAZ encoded protein and β-catenin transfected with si-YWHAZ or miR-218-5p inhibitors or both in MGC-803 and AGS. Scale bar, 20 µm. No significantly differences were presented as ns.

To further understand the involvement of the miR-218-5p/YWHAZ/β-catenin pathway in the regulation of the metastatic potential of GC cells induced by SNHG12, Transwell assays were performed in MGC-803 and AGS cells transfected with pCDH-CMV-SNHG12 and/or miR-218-5p mimics. Figures 5A, B show that the migrated cell count in MGC-803 and AGS cells transfected with pCDH-CMV-SNHG12 or miR-218-5p mimics was significantly increased or decreased, respectively. Nevertheless, when MGC-803 and AGS cells were co-transfected with pCDH-CMV-SNHG12 and miR-218-5p mimics, the increased in migrated cells induced by SNHG12 overexpression was reversed, and there was no significant difference from the control groups.

We further observed that the abnormal expression of EMT-related proteins, β-catenin and the YWHAZ-encoded protein induced by SNHG12 overexpression could be reversed after the introduction of miR-218-5p mimics (Figure 5C). Similarly, Transwell assays showed that the migrated cell count in MGC-803 and AGS cells transfected with sh-SNHG12/si-YWHAZ or miR-218-5p inhibitors was significantly decreased or increased, respectively. On the other hand, when MGC-803 and AGS cells were co-transfected with sh-SNHG12/si-YWHAZ and miR-218-5p inhibitors, the decreased in migrated cells induced by SNHG12 knockdown was reversed, and the results were not significantly different from those of the control groups (Figures 5D-H). Therefore, the abnormal expression of EMT-related proteins, β-catenin and the YWHAZ-encoded protein induced by SNHG12 knockdown was reversed after introduction of miR-218-5p inhibitors (Figures 5F, I).

### The transcription factor YY1 modulates SNHG12 expression

**Figure 6:**
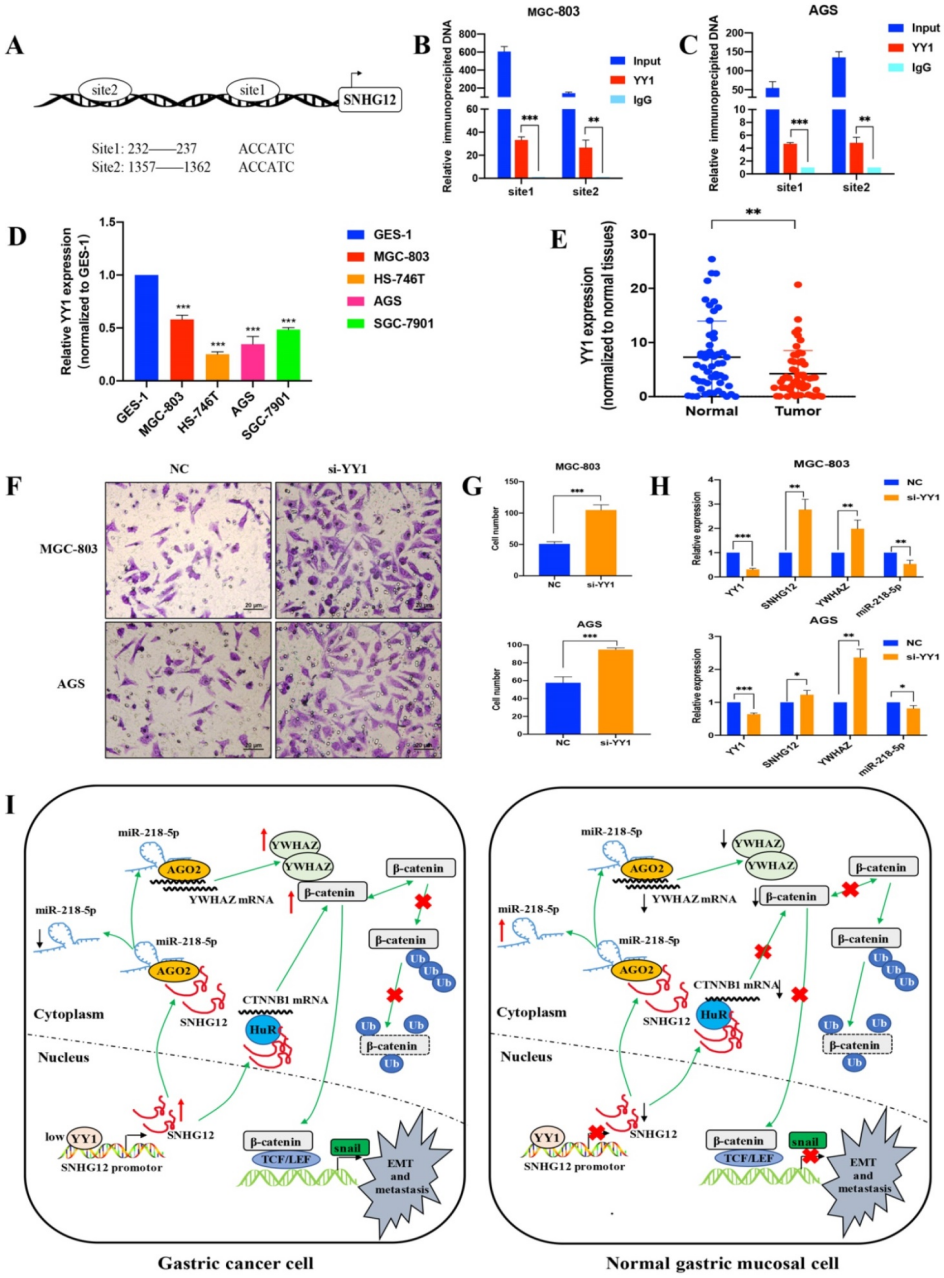
** Transcription factor YY1 regulates the expression of SNHG12. (A, B, C)** Bioinformatics analysis and CHIP assay showing YY1 binds to the promoter of SNHG12. **(D, E)** The expression of YY1 in GC tissues and cell lines. **(F, G)** Transwell assays showing the effects of the regulation of YY1 on GC metastasis. **(H)** qRT-PCR assays showing the expression of YY1, SNHG12, YWHAZ, miR-218-5p after YY1 knockdown. **(I)** Schematic illustration of the mechanism underlying SNHG12 regulation of GC metastasis and EMT. Scale bar, 20 µm. Significant results were presented as *P<0.05, **P<0.01, ***P<0.001.

To further elucidate the mechanism underlying SNHG12 overexpression in GC, we investigated the involvement of transcription factors in regulating the transcription of SNHG12. The JASPAR and PROMO databases were used to analyze the potential transcription factors that could bind to the SNHG12 promoter, and the transcription factor YY1 showed affinity for the binding site in the promoter of SNHG12. The Ch-IP assay results showed that the site 1 (+232 to +237) and site 2 (+1357 to +1362) regions in the SNHG12 promoter might mediate YY1 binding to the endogenous SNHG12 promoter (Figures 6A-C). qRT-PCR was performed to determine YY1 expression in the different GC cell lines and tissues. As shown in Figure 6D, compared with that in GES-1, YY1 expression in GC cell lines (MGC-803, AGS, HS-746T, and SGC-7901) was relatively low. Moreover, YY1 expression was significantly lower in GC tissue samples than that in the corresponding non-cancerous epithelial tissues (Figure 6E). The results from Transwell assays indicated that YY1 silencing led to a significant increase in the migration of MGC-803 and AGS cells (Figures 6F, G). Upon treatment with si-YY1, the expression of SNHG12 and YWHAZ increased, while that of miR-218-5p decreased (Figure 6H), which was verified by qRT-PCR. Thus, these results suggested that low YY1 expression in GC promotes the transcription of SNHG12.

## Discussion

Previous literature has outlined the role of SNHG12 in the tumorigenesis of several cancers, but its significance in GC requires further elucidation 9. In this study, we also confirmed that SNHG12 was highly expressed in GC cell lines and tissues and that its high expression was clinically closely related to the invasion depth, TNM stage and the extent of lymph node metastasis 12. On the other hand, ISH assays revealed that GC tissues showed much stronger SNHG12-positive staining than the adjacent non-cancerous epithelial tissues. Kaplan-Meier survival analysis confirmed that GC patients with a high SNHG12 expression profile had poorer DFS and OS rates than those with a low SNHG12 expression profile, and univariate and multivariate analysis demonstrated SNHG12 was an independent prognostic factor. Furthermore, increased expression of SNHG12 promoted GC cell metastatic potential *in vitro* and *in vivo*, indicating that SNHG12 is an important contributor to GC progression.

There have been many speculations about the mechanisms underlying the regulation of tumor progression by SNHG12. In this study, we clarified that SNHG12 is mainly located in the cytoplasm and sponges miR-218-5p-containing RISCs. For the first time, it has been shown that SNHG12 and miR-218-5p have a negative correlation in GC, and miR-218-5p can directly bind to the 3'UTR of SNHG12. The level of miR-218-5p expression in GC cell lines was low, suggesting that miR-218-5p can negatively regulate metastasis as a tumor suppressor. Further investigation revealed YWHAZ as a downstream target of both SNHG12 and miR-218-5p. YWHAZ expression is upregulated in multiple types of cancer, including GC, and YWHAZ has been identified as a potential biomarker for predicting the prognosis of GC patients 21, 22. Dual-luciferase reporter assays, Transwell assays and western blot assays with GC cell lines supported that the SNHG12/miR-218-5p/YWHAZ axis forms a competing endogenous (ceRNA) network and positively regulates GC metastasis and EMT; this is the first time these relationships among the three molecules have been revealed. Previous studies have demonstrated miR-218-5p could suppress migration and predict poor prognosis in various types of cancers, and miR-218-5p can also be sponged by many lncRNAs and circular RNAs (circRNAs) 13, 14, 23, 24. Thus, exploring why miR-218-5p is expressed at low levels in GC and whether it can regulate the microenvironment of GC metastases combination with SNHG12 and YWHAZ is warranted, and the results will be significant for GC diagnosis and therapy.

The Wnt/β-catenin signaling pathway is well-established to regulate cancer cell EMT and invasion. SNHG12 recruits IGF2BP2 to enhance the stability of CTNNB1, the gene encoding β-catenin 25. The YWHAZ-encoded protein (14-3-3ζ) is capable of activating the β-catenin pathway by inducing the accumulation of β-catenin in the cytosol and nucleus 26, 27. In this study, we discovered that SNHG12 could activate the β-catenin signaling pathway in GC cells by not only increasing the stability of CTNNB1 mRNA by binding with HuR but also regulating YWHAZ, which binds to β-catenin to reduce the ubiquitination-based degradation of β-catenin; these effects result in the overexpression of β-catenin, thus activating the downstream pathway and promoting metastasis and EMT, for instance, by activating TCF/LEF transcription elements. Snail, a direct target of the β-catenin/TCF complex, is known to activate the EMT program during cancer metastasis 28. Therefore, for the first time, we reveal additional novel mechanisms involving SNHG12 and β-catenin. Previous studies have showed that lncRNAs can stabilize some key proteins by regulating their ubiquitination 29, 30; thus, investigations are needed to determine whether SNHG12 can regulate the ubiquitination of YWHAZ and other cancer-related proteins, and the results will be significant for tumor therapy and diagnosis.

Loss of DNA methylation within the promoter region of SNHG12 makes it more accessible to Sp1, which regulates SNHG12 expression in glioblastoma 31. However, the mechanisms underlying SNHG12 overexpression in GC cells have not been elucidated. In our study, we discovered that YY1, which can regulate many lncRNAs as a transcription factor, was poorly expressed in GC and had the potential to inhibit SNHG12 transcription. We cannot exclude other mechanisms behind the transcriptional suppression of SNHG12 by YY1, as YY1 can also bind to HDACs, which inhibits histone acetylation 32, we speculate that YY1 may suppress the transcription of SNHG12 by directly binding to the SNHG12 promoter region and inducing epigenetic modification. However, this hypothesis requires further verification, and the detailed mechanisms of the transcriptional suppression of SNHG12 by YY1 remain to be discovered in the future. As the role of SNHG12 has been established in the GC via *in vivo*, *in vitro* and clinical analyses, a significant role of SNHG12 in diagnosis, targeted therapy and combination therapy is likely, and such studies have set the foundation for more clinical studies.

In conclusion, our findings demonstrate that SNHG12 is a potential prognostic marker and therapeutic target for GC. Negatively modulated by the transcription factor YY1, SNHG12 promotes GC metastasis and EMT by regulating the miR-218-5p/YWHAZ axis and stabilizing CTNNB1 mRNA, hence activating the β-catenin signaling pathway (Figure 6I).

## Supplementary Material

Supplementary figures.

## Figures and Tables

**Table A:** List of primers

SNHG12 F	TCTGGTGATCGAGGACTTCC
SNHG12 R	ACCTCCTCAGTATCACACACT
hsa-mir-195-5p F	ACGGTAGCAGCACAGAAATATTGGC
hsa-mir-218-5p F	AGCGGTTGTGCTTGATCTAACCATGT
hsa-mir-199a-5p F	GCCCAGTGTTCAGACTACCTGTTC
hsa-mir-199b-5p F	CGCGTCCCAGTGTTTAGACTATCTGTTC
β-catenin F	AAAGCGGCTGTTAGTCACTGG
β-catenin R	CGAGTCATTGCATACTGTCCAT
YWHAZ F	TTTCTCCTTCCCCTTCTTCCG
YWHAZ R	GCCAGTTTGGCCTTCTGAAC
GAPDH F	CCCATCACCATCTTCCAGGAG
GAPDH R	CTTCTCCATGGTGGTGAAGACG
β-actin F	CTCCATCCTGGCCTCGCTGT
β-actin R	GCTGTCACCTTCACCGTTCC
YY1 F	AGCAGAAGCAGGTGCAGATCAA
YY1 R	CTGCCAGTTGTTTGGGATCT

**Table B:** List of primers in Ch-IP assays

Site1 F	ATCGAGACCATCCTGCCCAAC
Site1 R	ACGGAGCTGCTCTGTCGC
Site2 F	CAGCAGGATCTGGGAGATAAGAGACT
Site2 R	CTGTGGTCCTGGACTCCTCTCT

**Table C:** List of primers in RIP

RIP-SNHG12-1F	CCTTCTCTCGCTTCGGACTG
RIP-SNHG12-1R	TTACCCCGGAAGTCCTCGAT
RIP-SNHG12-2F	ACAGGCGGATAAAACGGTCC
RIP-SNHG12-2R	AGTACGCCGGGATCTCTGTA
RIP-SNHG12-3F	GGGCCTACAGGATGACTGAC
RIP-SNHG12-3R	CAACCAGGTCCCCTGCATTT
RIP-SNHG12-4F	GGCTGACAGGCGGATAAAAC
RIP-SNHG12-4R	GTACGCCGGGATCTCTGTAG
RIP-YWHAZ-1F	ACTCCCGTTTCCGAGCCATA
RIP-YWHAZ-1R	CTCCAAGATGACCTACGGGC
RIP-YWHAZ-2F	AAAGGTCTAGGACCGCTTCC
RIP-YWHAZ-2R	CCAAGATGACCTACGGGCTC
RIP-YWHAZ-3F	CTCTCGATTGGAACGCCTCC
RIP-YWHAZ-3R	ACTGGATGTTCTGCTGGCTC
RIP-YWHAZ-4F	CCATCACTCAGCCACACTCA
RIP-YWHAZ-4R	GGCCTTCTGAACCAGCTCAT

**Table 1:** Correlation between the clinicopathological features and expression of SNHG12

Clinical parameter	SNHG12 expression		*P* value
	High expression cases (n=36)	Low expression cases (n=18)	
**Gender**			0.837
Male	25	12	
Female	11	6	
**Age (years)**	65.3±10.2	62.6±10.7	0.363
**Tumor Size (tumor maximum axis)**			0.126
≥5 cm	22	7	
<5 cm	14	11	
**Borrmann Type**			0.105
Type I	4	1	
Type II	12	12	
Type III	19	5	
Type IV	1	0	
**Histologic differentiation**			0.344
Well and moderate	7	5	
Poor	29	13	
**T Stage**			<0.0001
T1/T2	5	9	
T3/T4	31	9	
**N Stage**			<0.0001
N0	5	11	
N1	6	2	
N2	7	4	
N3a	7	1	
N3b	11	0	
**TNM Stage**			<0.0001
I	1	5	
IIA	4	3	
IIB	4	8	
IIIA	4	1	
IIIB	7	0	
IIIC	16	1	
**LNM Rate**	25.6%±26.0%	6.7%±13.3%	0.007

NOTE: TNM, tumor node metastasis; LNM, lymph node metastasis; p<0.05 significant result.

**Table 2:** Univariate and multivariate Cox regression analysis of SNHG12 and disease-free survival in patients with gastric cancer

Variables	5-year DFS rate	Univariate	Multivariate		
		*P* value	HR	95%CI	*P* value
**Gender**		0.052			
Male	54.10%				
Female	94.40%				
**Age (years)**		0.396			
≥60	59.00%				
<60	66.70%				
**Tumor Size**		0.153			
≥5 cm	55.20%				
<5 cm	68.00%				
**Histologic differentiation**		0.596			
Well and moderate	63.60%				
Poor	60.50%				
**T Stage**		0.033			
T1/T2	85.70%				
T3/T4	52.50%				
**N Stage**		0.081			
N0/N1	79.20%				
N2/N3	46.70%				
**TNM Stage**		0.005			
I/II	84.00%				
III/IV	41.40%				
**SNHG12 expression**		0.001	0.069	0.009-0.516	0.009
High	44.40%				
Low	94.40%				

NOTE: DFS, disease-free survival; HR, hazard ratio; CI, confidence interval; TNM, tumor node metastasis; p<0.05 significant result.

**Table 3:** Univariate and multivariate Cox regression analysis of SNHG12 and overall survival in patients with gastric cancer

Variables	5-year OS rate	Univariate	Multivariate		
		*P* value	HR	95%CI	*P* value
**Gender**		0.035			
Male	59.50%				
Female	82.40%				
**Age (years)**		0.168			
≥60	61.50%				
<60	80.00%				
**Tumor Size**		0.168			
≥5 cm	62.10%				
<5 cm	72.00%				
**Histologic differentiation**		0.714			
Well and moderate	67.40%				
Poor	63.60%				
**T Stage**		0.052			
T1/T2	85.70%				
T3/T4	60.00%				
**N Stage**		0.188			
N0/N1	79.20%				
N2/N3	56.70%				
**TNM Stage**		0.014			
I/II	84.00%				
III/IV	51.70%				
**SNHG12 expression**		0.002	0.078	0.010-0.593	0.014
High	52.80%				
Low	94.40%				

NOTE: OS, overall survival; HR, hazard ratio; CI, confidence interval; TNM, tumor node metastasis; p<0.05 significant result.

## Abbreviations

YY1: Ying Yang 1
lncRNA: long non-coding ribonucleic acid
SNHG12: small nucleolar RNA Host Gene 12
GC: gastric cancer
YWHAZ: tyrosine 3-monooxygenase/tryptophan5-monooxygenase activation protein, 14-3-3 protein zeta
EMT: epithelial-mesenchymal transition
qRT-PCR: reverse transcription- quantitative polymerase chain reaction
Ch-IP: chromatin immunoprecipitation
DFS: disease free survival
OS: overall survival
HuR: Hu antigen R
ncRNA: non-coding ribonucleic acid
miRNA: micro ribonucleic acid
shRNA: short hairpin ribonucleic acid
siRNA: small interfering RNA
FISH: fluorescence *in situ* hybridization
IHC: immunohistochemistry
ISH: *in situ* hybridization
BSA: bovine serum albumin
RIP: RNA-binding immunoprecipitation
ceRNA: competing endogenous ribonucleic acid
RBP: RNA-binding protein
TCF/LEF: T-Cell Factor/Lymphoid Enhancer Factor
