# Supplementary material for: YY1-modulated long non-coding RNA SNHG12 promotes gastric cancer metastasis by activating the miR-218-5p/YWHAZ axis
Source: Int J Biol Sci. 2021 Apr 12;17(7):1629–43. doi: 10.7150/ijbs.58921 (PMC8120461; doi:10.7150/ijbs.58921)

Additional file 1

Figure S1. Bioinformatics analysis and experimental verification suggest that SNHG12 could regulate miR-218-5p. **(a)** Using bioinformatics databases (Starbase and miRcode), 14 miRNAs related to SNHG12 were identified. **(b)** According to the gastric cancer data in Starbase online databases, the correlation of the 14 miRNAs with SNHG12 were analyzed, and miR-199a-5p, miR-199b-5p, miR-195-5p and miR-218-5p were identified as negatively related to SNHG12 with low expression profile in GC (r refers to the correlation coefficient in Starbase online databases). **(c, d)** SNHG12 knockdown in MGC-803 and AGS revealed that the change in the expression of miR-218-5p was most significant among the 4 miRNAs.

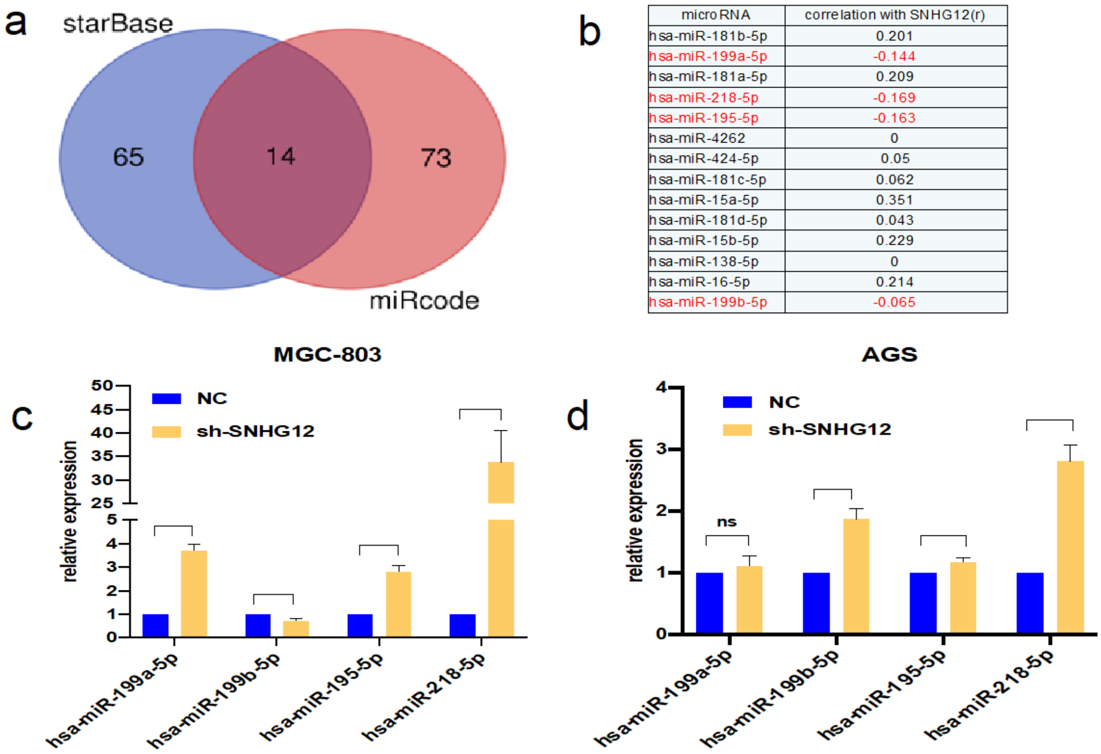

Figure S2. Bioinformatics analysis indicated the target gene of miR-218-5p and SNHG12. (a) Using the databases TargetScan Human 7.2 and Starbase, 6 mRNAs were identified as the putative downstream genes of SNHG12 and miR-218-5p. (**b)** According to th GC data in Starbase online databases, the correlations of the 6 identified genes with SNHG12 and miR-218-5p were analyzed,, YWHAZ and RCC1 displayed high positive correlation with SNHG12, negative correlation with miR-218-5p, while YWHAZ was closely related with tumor metastasis. (**c, d, e)** The correlations between SNHG12, miR-218-5p and YWHAZ from the gastric cancer data in Starbase online databases.

a

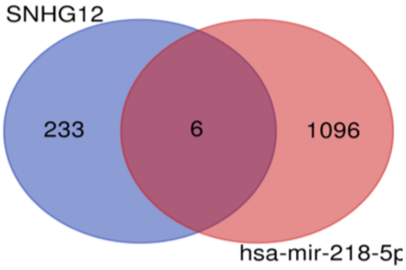

b

mRNA	correlation with SNHG12 (r)	correlation with miR-218-5p (r)
YWHAZ	0.342	-0.384
SLC5A3	0.108	-0.141
RCC1	0.417	-0.259
BAZ2B	0.235	0.009
DDX6	0.072	0.052
ONECUT2	0.095	-0.065

c

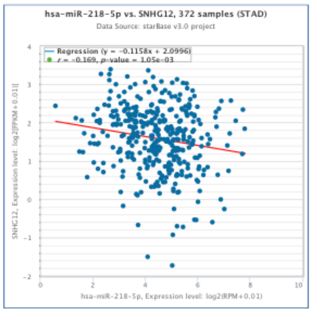

d

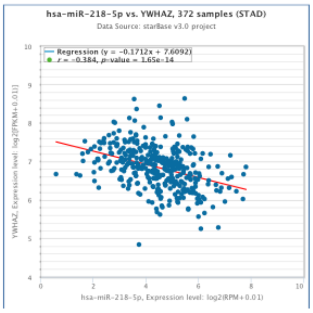

e

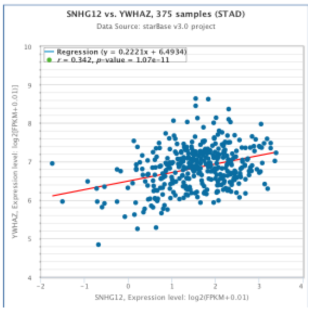

Supplement: Supplementary file 1 — Supplementary figures. [file ijbsv17p1629s1.pdf]
